# Case Report: Chronic Bacterial Prostatitis Treated With Phage Therapy After Multiple Failed Antibiotic Treatments

**DOI:** 10.3389/fphar.2021.692614

**Published:** 2021-06-10

**Authors:** Apurva Virmani Johri, Pranav Johri, Naomi Hoyle, Levan Pipia, Lia Nadareishvili, Dea Nizharadze

**Affiliations:** ^1^ Vitalis Phage Therapy, New Delhi, India; ^2^ Eliava Phage Therapy Center, Tbilisi, Georgia

**Keywords:** phage therapy, chronic bacterial prostatitis, bacteriophages, antibiotic resistance, biofilm, case report

## Abstract

**Background:** Chronic Bacterial Prostatitis (CBP) is an inflammatory condition caused by a persistent bacterial infection of the prostate gland and its surrounding areas in the male pelvic region. It is most common in men under 50 years of age. It is a long-lasting and debilitating condition that severely deteriorates the patient’s quality of life. Anatomical limitations and antimicrobial resistance limit the effectiveness of antibiotic treatment of CBP. Bacteriophage therapy is proposed as a promising alternative treatment of CBP and related infections. Bacteriophage therapy is the use of lytic bacterial viruses to treat bacterial infections. Many cases of CBP are complicated by infections caused by both nosocomial and community acquired multidrug resistant bacteria. Frequently encountered strains include Vancomycin resistant *Enterococci*, Extended Spectrum Beta Lactam resistant *Escherichia coli*, other gram-positive organisms such as *Staphylococcus* and *Streptococcus*, *Enterobacteriaceae* such as *Klebsiella* and *Proteus*, and *Pseudomonas aeruginosa*, among others.

**Case Presentation:** We present a patient with the typical manifestations of CBP. The patient underwent multiple courses of antibiotic treatment without any long-term resolution of his symptoms. Testing of prostatic secretion and semen samples revealed pathogenic bacteria in each case, which collectively included members of the Staphylococcal species such as Methicillin resistant *Staphylococcus aureus* (MRSA) and *Staphylococcus haemolyticus*, *Enterococcus faecalis*, and *Streptococcus mitis*, among others.

**Methods and Outcome:** Bacteriophage preparations from the Eliava Institute were used to treat the patient after establishing phage sensitivity to the pathogenic bacteria. Significant improvements in symptoms and re-testing of samples after bacteriophage treatment indicated a reduction in the bacterial load and resolution of the infection.

**Discussion:** The patient saw significant improvement of symptoms, and positive dynamics in bacterial titers and ultrasound controls after phage therapy. The failure of antibiotic therapy and subsequent success of bacteriophage therapy in treating chronic bacterial prostatitis shows the effectiveness of bacteriophages in controlling chronic infections in areas of low vascularity and anatomical complexity. These cases also highlight the efficacy of phages in overcoming antibiotic-resistant infections as well as biofilm infections.

## Introduction

### About the Disease: Chronic Bacterial Prostatitis

Chronic Bacterial Prostatitis is an inflammatory condition caused by persistent bacterial infection of the prostate gland and surrounding areas in the male pelvic region (Krieger, et al., 2008). The United States National Institutes of Health classify prostatitis into four internationally accepted categories: Category I—Acute Bacterial Prostatitis (ABP); Category II—Chronic Bacterial Prostatitis (CBP); Category III—Chronic Prostatitis/Chronic Pelvic Pain Syndrome (CP/CPPS); Category IV—Asymptomatic Inflammatory Prostatitis (AIP) (Krieger, et al., 1999). Estimates suggest that prostatitis afflicts from 2–16% of all men worldwide, with a recurrence rate of up to 50% (Roberts, et al., 1998; Krieger, 2004; Krieger, et al., 2008).

CBP is diagnosed by the presence of symptoms, examination of the prostate, and lab tests to determine the bacterial nature of the condition.

Symptoms of CBP are usually prolonged. They can include:

(1) Urinary symptoms like urethral burning, difficulty starting a stream, urgency or increased frequency, nocturia, dribbling, and incomplete voiding of bladder;
(2) Pain in the perineum, suprapubic region, lower back, abdomen, penis, testicles, groin, and rectum, and pain during ejaculation and dysuria; and
(3) Sexual dysfunction, including erectile dysfunction, ejaculatory discomfort, hematospermia, and decreased libido.

Along with these symptoms, CBP is often accompanied by recurrent urinary tract infections, urethritis or epididymitis (Lipsky, et al., 2010; Sharp, et al., 2010; Bowen, et al., 2015; Rees, et al., 2015).

Since 1968, the standard diagnostic test to detect pathogens causing CBP is the Meares-Stamey “4-glass test” (Magri, et al., 2009). This involves the collection and testing of four samples: first catch urine—urethral specimen, midstream urine—bladder specimen, expressed prostatic secretion (EPS) and voided urine after EPS expression (Sharp, et al., 2010).

Presence of leukocytes along with positive cultures of the EPS or post-prostatic massage urine samples are considered necessary for a positive diagnosis of CBP. However, this is a time-consuming and costly process, rarely conducted by urologists. Primary care physicians and urologists often treat CBP empirically with antibiotics (McNaughton Collins, et al., 2000). In cases where tests are conducted, the simplified “2-glass test” is preferred to the Meares-Stamey 4-glass test. It entails cultures and microscopic examination of urine samples collected pre and post-prostatic massage (Nickel, et al., 2006; Sharp, et al., 2010). Recent research has also shown that bacteriological analysis of semen samples can accurately detect the pathogenic bacteria causing CBP (Budia, et al., 2006; Magri, et al., 2009). Semen analysis can complement but not replace the 4-glass test.

### About the Treatment: Bacteriophage Therapy

Bacteriophages, or simply phages, are bacterial viruses that are natural predators of bacteria. They are the most abundant entity in the world, outnumbering the bacterial cells in nature by a ratio of approximately 10:1, and are present in every environment that has bacteria. Phages are extremely specific, infecting and killing only their particular strains of bacteria. (Clokie, et al., 2011).

Bacteriophage therapy is the application of lytic phages for therapeutic purposes, i.e., to infect and destroy colonies of bacterial pathogens (Koskella and Meaden, 2013; Chanishvili, 2016). Lytic phages propagate by injecting their DNA into the bacterial cell, disrupting bacterial metabolism and replicating inside the cell. These progeny phages then lyse the bacterial cell and are released to infect an exponentially higher number of bacterial cells of the same strain or colony, and the process repeats itself (Guo, et al., 2020).

Treatment of bacterial infections with phages was explored across the world before the advent of antibiotics. Phages were first discovered in 1917 and are widely used in Eastern European countries such as Georgia, Poland, and Russia. The George Eliava Institute of Bacteriophages, Microbiology, and Virology in Tbilisi, Georgia was founded in 1923 by George Eliava, a Georgian microbiologist, along with Felix d’Herelle, the French-Canadian scientist who discovered phages (Chanishvili, 2016).

The institute scientists established a clinic, the Eliava Phage Therapy Center (EPTC), to specialize in bacteriophage therapy in 2011. Since, patients with antibiotic resistant infections have traveled to Tbilisi for treatment from nearly 70 countries worldwide.

The use of phage therapy as an alternative treatment in CBP is a longstanding practice of both the EPTC in Georgia as well as in the Hirszfeld Institute of Immunology and Experimental Therapy in Poland (Letkiewicz, et al., 2010; Górski, et al., 2018; Ujmajuridze, et al., 2018). Urological conditions including cystitis, chronic urinary tract infections and CBP are some of the most frequently treated conditions at the EPTC (Kuipers, et al., 2019; Corbellino, et al., 2020). Scientists from the Eliava Institute collaborated with Swiss colleagues to study phage therapy as a method for reducing bacterial infection after transurethral resection of the prostate. This is the only double-blind clinical trial of phage therapy in urology to date (Leitner, et al., 2017; Leitner, et al., 2021).

### Antibiotic Resistance and Phage Therapy

Antibiotic resistance is a growing problem across the world, as bacteria rapidly evolve to develop resistance to antibiotics currently in use globally. According to a United Kingdom Department of Health study, by the year 2050, 10 million people will die every year due to bacterial infections that are not treatable with antibiotics. This number puts the estimated deaths due to antibiotic-resistant infections to be higher than cancer (O’Neill, 2016).

Phage therapy is one of the key alternatives to antibiotics suggested in the O’Neill review (O’Neill, 2016). As multidrug-resistant “superbug” bacteria emerge and the crisis of antibiotic resistance grows, there is a renewed interest in phage therapy amongst scientists, researchers and public health administration bodies globally (Kutter, et al., 2015; Abedon, et al., 2017). Various countries have given approvals for clinical trials and compassionate use of bacteriophages over the last two years (Phagoburn, 2017; Pirnay, et al., 2018; Voelker, 2019).

### Advantages of Phage Therapy for Chronic Infections

Phages have numerous advantages in the treatment of chronic bacterial infections such as CBP. In contrast with antibiotics, phages are bactericidal, have a narrow host range, are self-replicating, adapt to bacterial resistance, penetrate biofilms, and have minimal side effects even with long term usage, as is typically required for antibacterial therapy in chronic bacterial infections (Carlton, 1999; Loc-Carrillo and Abedon, 2011; Pires, et al., 2017; Hoyle and Kutter, 2021). Additionally, they can be used in conjunction with antibiotics for synergistic impact on clearing bacterial pathogens. Resistance to one can make bacteria more sensitive to the other. This phage-antibiotic synergy (PAS) makes them especially useful for treating multidrug-resistant superbugs (Comeau, et al., 2007).

In addition, recent studies show that along with bactericidal action, phages also have immune modulating effects, primarily anti-inflammatory effects with chronic inflammatory conditions like CBP; phage therapy holds the potential to provide infection control as well as inflammation reduction. This reduces future probability of development of conditions caused by chronic inflammation, such as cancer (Górski, et al., 2018).

## Presentation of the Case

A 33-year-old Indian male had the following subjective symptoms from June 2016 till November 2016: Sharp pain in the right testicle radiating to the right buttock, right lower back, pelvic region both left and right sides, and perineal pain. He also experienced perspiration, generalized weakness and malaise in the body through the day.

The patient had a daily low-grade fever and chills: 37.5–37.7°C. No antipyretic was taken to reduce body temperature. He felt chills every morning that would last for about 1.5 h.

At this time, a urine culture was ordered, which was sterile after 48 h of aerobic incubation. A kidney, ureter, and urinary bladder (KUB) ultrasound showed both kidneys to be normal in size, shape, position, and echotexture. No evidence of any calculus or hydronephrosis was noted. The urinary bladder was normally distended with normal wall thickness. No calculus was observed. The prostate gland was considered to be of normal size.

A digital rectal exam (DRE) by a urologist revealed a tender prostate, and the patient was diagnosed with CBP. He had no history of urological problems before this diagnosis. Multiple antibiotic treatments were administered empirically in the patient’s home country over a period of four months. These included single dose Azithromycin 1 g, followed by a course of Doxycycline 200 mg for 10 days, then the third course of antibiotics with Ofloxacin 400 mg for 23 days, and finally a combination of Ciprofloxacin 1 g taken orally and Amikacin 750 mg given intravenously for 10 days.

The patient experienced no improvement in symptoms during or after these antibiotic courses.

A transrectal ultrasound (TRUS) done in October 2016 showed the prostate size to be 21.98 ml.

In November 2016, the patient traveled to Tbilisi, Georgia, to explore phage therapy at the EPTC as a potential treatment for his condition. At the clinic, a full urologic workup was performed. The patient’s prostate was found to be tender and boggy by rectal palpation. The patient’s EPS and semen samples were collected and observed microscopically, as well as cultured for aerobic bacteria. The cultures were tested for sensitivity against Eliava Institute’s standard phage cocktail preparations. The details of these phage preparations are given in Appendix Table A1. Table 1 shows the outcomes of these tests.

**Table 1:** Results of analysis and cultures of fluids from the infected region—November 2016.

Specimen name	Leukocyte count	Bacteria cultured	Growth	Phages preparations showing bactericidal action
EPS	50–60 / FoV (Field of vision)	*Staphylococcus haemolyticus*	>1 × 10^8^ CFU/ml	Pyo bacteriophage
				Intesti bacteriophage
				Staphylococcal bacteriophage
		*Staphylococcus aureus*	<1 × 10^3^ CFU/ml	Pyo bacteriophage
				Intesti bacteriophage
				Ses bacteriophage
				Enko bacteriophage
				Staphylococcal bacteriophage
Semen	15–20 / FoV	*Enterococcus faecalis*	<1 × 10^3^ CFU/ml	Intesti bacteriophage
		*Staphylococcus epidermidis*	<1 × 10^3^ CFU/ml	Pyo bacteriophage
				Staphylococcal bacteriophage

No fungal growth was detected in either sample. There was no presence of gonococcus. Blood tests showed normal blood counts, leukocyte counts, erythrocyte sedimentation rate (ESR), C-reactive protein (CRP) and prostate-specific antigen (PSA) levels. Urinalysis and culture were sterile.

*S. epidermidis* was considered non-pathogenic due to its low growth and low virulence.

The patient decided to undergo phage therapy. Pyo, Intesti, and Staphylococcal phage preparations were used for his treatment. The preparations were administered in three forms—oral liquid, rectal suppositories, and urethral instillations.

20 ml each of Pyo and Intesti oral phages were given to the patient per day for the first 14 days. Concurrently, the patient self-administered Staphylococcal phage suppositories twice a day for 10 days, and urethral instillations with Intesti phage were administered to him by the urologist at the EPTC once a day for 10 days.

On the fifth day after starting phage therapy, the patient’s body temperature normalized, and did not subsequently increase beyond 37°C.

After the first 2 weeks, a long-term daily dose of 10 ml each of Pyo and Intesti oral phages was established for the next 2 months. Rectal suppositories of Pyo, Intesti, and Staphylococcal bacteriophages were used in rotation for 10 days each, with breaks of 10 days between different phage suppositories. Urethral instillations were not done after the initial 10 days to avoid urethral irritation.

The testicular and back pains increased initially after starting treatment and began to subside after 3 weeks of starting phage therapy. Subjective symptoms of weakness, night sweating, and chills also decreased gradually.

In March 2017, the patient visited the EPTC again, and his EPS and semen were tested to ascertain his progress. Table 2 shows the outcomes of the tests.

**Table 2:** Results of analysis and cultures of fluids from the infected region—March 2017.

Specimen name	Leukocyte count	Bacteria cultured	Growth	Phages preparations showing bactericidal action
EPS	30–40 / FoV	*Staphylococcus haemolyticus*	>1 × 10^8^ CFU/ml	Intesti bacteriophage
				Fersis bacteriophage
		*Streptococcus mitis*	>1 × 10^8^ CFU/ml	None
Semen	10–15 / FoV	*Enterococcus faecalis*	<1 × 10^3^ CFU/ml	Intesti bacteriophage
		*Staphylococcus epidermidis*	<1 × 10^3^ CFU/ml	Intesti bacteriophage
				Staphylococcal bacteriophage

*S. aureus* did not grow in this or any subsequent cultures. *S. mitis* was a new bacterium that grew in the EPS. This strain was resistant to all of Eliava Institute’s standard phage preparations. The other bacteria were treated with Intesti and Fersis phage preparations from March till June 2017. A combination of oral phage, rectal suppositories, and urethral instillations was administered, similar to the previous course of treatment.

Through this course of treatment, the patient’s symptoms continued to improve. Night sweats, chills, excessive perspiration, and weakness had fully subsided by the end of June 2017.

A custom phage (autophage) was prepared in September 2017 that was fully sensitive against the *S. mitis* isolated from the patient’s sample. This was administered according to the previous protocol, along with Staphylococcal bacteriophage, from November 2017 till January 2018, during which time his only remaining symptoms of pelvic and perineum pain decreased in intensity and frequency.

**FIGURE 1:**
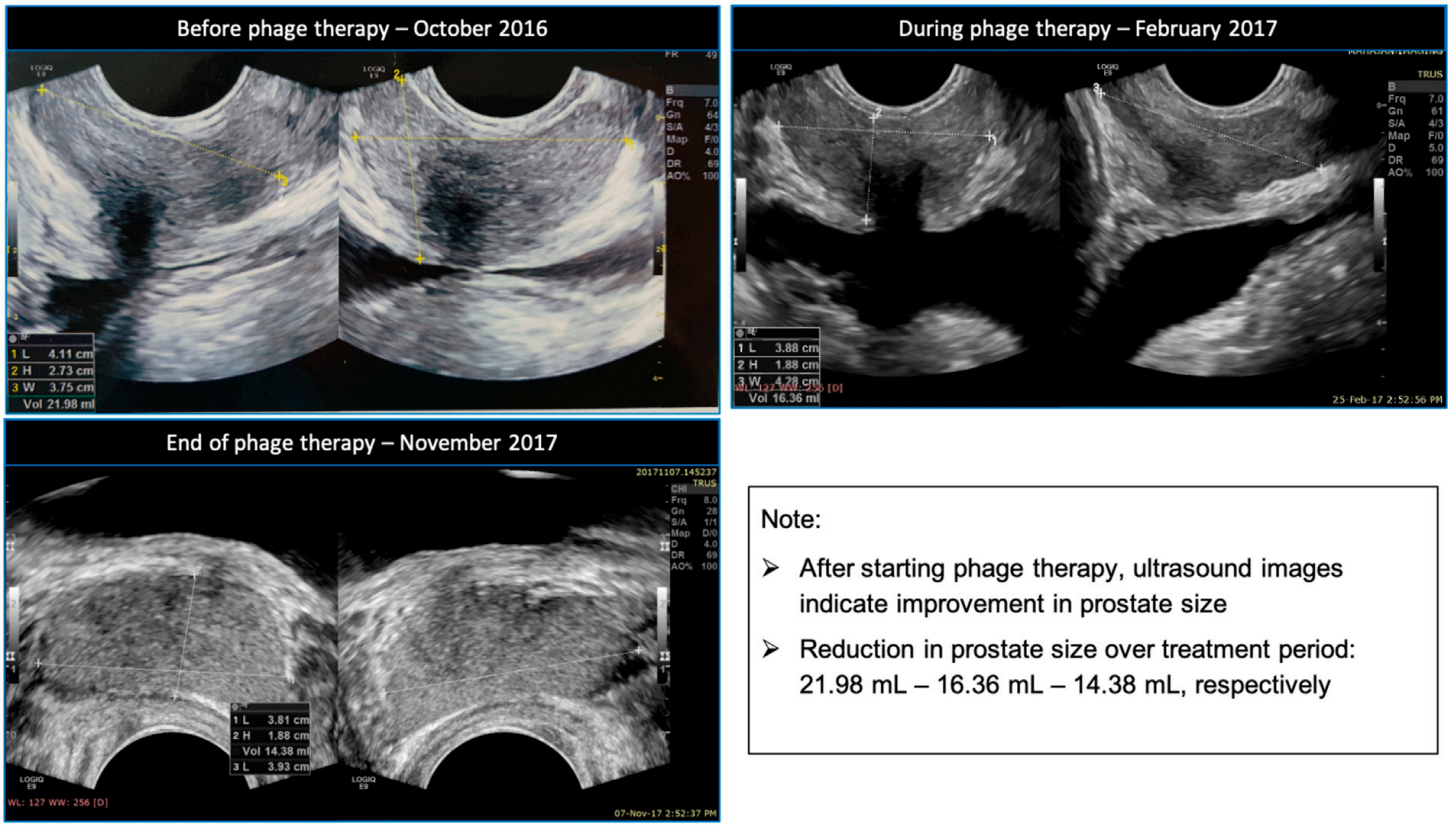
Clockwise ultrasound images of the patient’s prostate before, during and towards the end of his phage therapy.

A TRUS performed in November 2017 revealed that the prostate size had reduced to 14.38 ml, with no prostatic inflammation present. Figure 1 shows a comparison between the ultrasound images before, during and towards the end of the patient’s phage therapy.

The patient’s EPS and semen were tested again in May 2018. No pathogenic bacteria grew in these cultures, and the leukocyte counts in the EPS and semen were normal. The prostate was small and firm by rectal palpation. Repeated testing has continued to show the same results. The patient is in full remission, and his chief complaints have not returned.

## Discussion

The challenges of treating CBP are well known in the medical community. CBP is known to significantly impair the quality of life of the sufferer. Patients score poorly on tests of both physical and mental health parameters. The reduction in quality of life is comparable with that of patients suffering from congenital heart failure and diabetes mellitus (McNaughton Collins, et al., 2001).

Oral antimicrobial agents are commonly used to treat CBP, chief among them fluoroquinolones, tetracyclines, macrolides, and trimethoprim-sulfamethoxazole. (Sharp, et al., 2010; Bowen, et al., 2015; Rees, et al., 2015). Most drugs in these classes of antibiotics have high lipid solubility and favourable diffusion values through the lipid membrane of the prostatic epithelium. They have been shown to achieve minimum inhibitory concentration (MIC) in the prostatic secretion (Charalabopoulos, et al., 2003).

Recurrence of CBP is common after treatment with antibiotics. In many cases, despite taking antibiotics with good absorption into the prostate, patients continue to have symptoms. This is possibly due to biofilm formation and antibiotic resistance of the pathogenic bacteria (Mazzoli, 2010; Wagenlehner, et al., 2014). Biofilms are at the root of many chronic bacterial infections, including CBP (Costerton, et al., 1999). It is difficult for widely used antimicrobials to eradicate such infections, as bacterial cells residing within biofilms can be highly resistant to antibiotics as compared to planktonic cells of the same bacteria (Mah and O’Toole, 2001; de la Fuente-Núñez, et al., 2013). Additionally, prostatic calcifications may accompany some CBP cases and are linked with biofilm formation and biofilm-producing bacteria (Mazzoli, 2010).

Phage therapy is a promising new approach for the treatment of CBP and related conditions, with patients from around the world seeking treatment with bacteriophage (Su, et al., 2020). The well-documented bactericidal, anti-biofilm and anti-inflammatory effects of therapeutic phages have no doubt augmented this trend (Carlton, 1999; Pires, et al., 2017; Górski, et al., 2018; Hoyle and Kutter, 2021).

The patient described in this case study underwent numerous courses of antibiotics without improvement in symptoms or adequate assessment such as bacteriologic analysis and TRUS. This failure of antibiotics in providing clinical improvement led to his pursuit of an alternative treatment. Phage therapy showed efficacy in both eradication of pathogenic bacteria as observed in repeat microbiological analyses and reduction in inflammation in the prostate as well as volume without burdening the patient with side effects. The quality of life of the patient improved drastically. He is now symptom free and has restoration of normal activity.

In this case, treatment courses with antibiotics in the patient’s home country may not have been adequate, with the exception of the 33-day fluoroquinolone therapy. In order to claim this was a case of antibiotic failure, administration of more than one cycle of antibiotic therapy following international guidelines of dosage and timing would have excluded responsiveness of the patient to standard treatment (Magri, et al., 2007; Lipsky, et al., 2010; Kraemer, et al., 2019). The lack of more than one antibiotic therapy administered as per such guidelines prior to the phage therapy represents a limitation to this report.

Phage therapy is a viable treatment method for patients suffering from bacterial infections untreatable with antibiotics due to bacterial resistance, antibiotic allergy, or undesirable side effects of long-term use of antibiotics. Phage therapy can be employed as a substitute to antibiotics for treating chronic infections, while using antibiotics for more acute or emergent infections. The Eliava Institute has six standard phage cocktail preparations against specific bacterial strains (see Appendix Table A1). When a bacterial pathogen becomes resistant to the standard phage preparations, or if there is an infection caused by bacteria other than the ones targeted by the standard phage preparations, a customised monophage preparation against the patient’s strain can be prepared. Standard phages have the advantage of being polyvalent cocktail preparations, making it more difficult for bacteria to develop resistance to them, as opposed to the customised monophage preparations which are adapted to the target bacteria through serial passage. (Pirnay, et al., 2011; Rhode, et al., 2018).

Phages are applied via three routes in the case of CBP at the EPTC–oral, rectal and urethral. The oral route provides systemic distribution of the phages, while local phage application *via* the rectal and urethral routes is known to be an efficient method of phage delivery to the infected region, in this case the prostate gland (Letkiewicz, et al., 2010; Qadir, Mobeen, and Masood, 2018).

Use of phages is known to alter the antibiotic resistance of bacteria—as bacteria evolve to resist phage action, they may become more sensitive to certain antibiotics, as antibiotics and phages have different mechanisms of action against bacteria (Hanlon, 2007). PAS can be a useful method to eradicate bacterial colonies and treat bacterial infections (Comeau, et al., 2007; Liu, et al., 2020).

With the growing threat of antibiotic resistance around the world, research in novel treatments for bacterial infections such as phage therapy is the need of the hour. Interest in phage research and therapeutics has been growing rapidly around the globe. A few countries have allowed therapeutic use of phages in a regulated manner. Continuing targeted research would allow more countries to adopt this treatment methodology for infection control. For this, controlled studies are needed to establish safety and efficacy data, and the parameters for beneficial use of this treatment protocol. We hope that case reports of patients recovering from chronic bacterial infections by undergoing phage therapy would provide valuable data to researchers around the world, and further their conviction to pursue research in this field.

## Data Availability

The original contributions presented in the study are included in the article/Supplementary Material, further inquiries can be directed to the corresponding author.

## Appendix A

Details of the standard phage preparations made by the Eliava Institute are in Table A1.

**TABLE A1:** Standard Phage Preparations made by the Eliava Institute of Bacteriophages, Microbiology and Virology.

Phage preparation	Composition	Titer: PFU (plaque-forming unit)/ml
Pyo bacteriophage	Phage lysates of *Streptococcus* spp., *Staphylococcus* spp., *Escherichia coli*, *Pseudomonas eruginosa*, and *Proteus* spp.	≈10^5^
Intesti bacteriophage	Phage lysates of *Staphylococcus* spp., *Enterococcus* spp., *Proteus* spp., *Shigella* spp., *Salmonella* spp., *Escherichia coli*, and *Pseudomonas eruginosa*	≈10^5^
Fersis bacteriophage	Phage lysates of *Staphylococcus* spp., and *Streptococcus* spp.	≈10^5^
Ses bacteriophage	Phage lysates of *Staphylococcus* spp., *Streptococcus* spp., and *Escherichia coli*	≈10^5^
Enko bacteriophage	Phage lysates of *Staphylococcus* spp., *Shigella* spp., *Salmonella* spp., and *Escherichia coli*	≈10^5^
Staphylococcal bacteriophage	Phage lysates of *Staphylococcus* spp.	≈10^7^
